# circRNA_0001006 predicts prognosis and regulates cellular processes of triple-negative breast cancer via miR-424-5p

**DOI:** 10.1186/s13008-023-00089-4

**Published:** 2023-05-16

**Authors:** Jiaqi Liu, Linna Kong, Wenqing Bian, Xiaona Lin, Feifei Wei, Jun Chu

**Affiliations:** 1 Department of Breast and Thyroid Surgery, Zibo Central Hospital, 54 Gongqingtuan West Road, Zibo, 255020 China; 2 Maternal and Child Health Hospital of Zibo, Zibo, 255095 China; 3 Weifang Maternal and Child Health Hospital, Weifang, 261000 China

**Keywords:** TNBC, Prognosis, Tumor progression, circRNAs, miRNAs

## Abstract

**Background:**

circular RNAs (circRNAs) have been considered novel biomarker candidates for human cancers, such as triple-negative breast cancer (TNBC). circ_0001006 was identified as a differentially expressed circRNA in metastatic breast cancer, but its significance and function in TNBC were unclear. The significance of circ_0001006 in TNBC was assessed and exploring its potential molecular mechanism to provide a therapeutic target for TNBC.

**Results:**

circ_0001006 showed significant upregulation in TNBC and close association with patients’ histological grade, Ki67 level, and TNM stage. Upregulated circ_0001006 could predict a worse prognosis and high risk of TNBC patients. In TNBC cells, silencing circ_0001006 suppressed cell proliferation, migration, and invasion. In mechanism, circ_0001006 could negatively regulate miR-424-5p, which mediated the inhibition of cellular processes by circ_0001006 knockdown.

**Conclusions:**

Upregulated circ_0001006 in TNBC served as a poor prognosis predictor and tumor promoter via negatively regulating miR-424-5p.

## Background

According to the latest data, the incidence of breast cancer has jumped to the top globally. Due to the changing habits of humans, drinking, smoking, obesity, and stress all contribute to the progressing development of breast cancer. The incidence of breast cancer is gradually increasing, and the age of onset is getting younger [1, 2]. Recently, developed screening methods dramatically benefit the early detection and therapies of breast cancer, but resistance and recurrence remain critical barriers resulting in an unsatisfactory prognosis. According to the status of ER, PR, HER-2, and Ki67, breast cancer was divided into four subtypes: Luminal A, Luminal B, HER2-positive, and triple-negative. The incidence, mortality, survival rate, and therapeutic effect of different subtypes are distinct, where triple-negative breast cancer (TNBC) was with the highest malignancy and worst outcome [3, 4]. The lack of effect biomarkers revealing the development of TNBC results in limited and unsatisfying therapeutic effects [5].

Molecular targets provide the basis for drug development involving susceptible genes and their products, signaling-related transcription factors, non-coding RNAs (ncRNAs), and other molecules. With the in-depth research on ncRNAs, microRNAs (miRNAs) and circular RNAs (circRNAs) are closely associated with TNBC development and are of great potential to serve as therapeutic targets. Different from linear RNAs, circRNAs have no 5′ or 3′ polyadenylate tails and are a series of closed circular RNA molecules formed by covalent bonds [6, 7]. Compared with linear RNAs, circRNAs are more stable because their structures are difficult to be hydrolyzed by exonuclease enzymes. Although the mechanism underlying the biological function of circRNAs remains unclear, there have been several circRNAs being suggested to mediate tumor progression and predict disease development of TNBC. circRNAs could act as sponges or bait of miRNAs protecting mRNA from the degradation by miRNA, and therefore facilitate the activity of functional genes indirectly [8, 9]. In a previous circRNA profile of breast cancer metastasis, several key circRNAs–miRNAs pairs were identified, where circRNA_0001006 was included [10]. In mechanism, miR-424-5p was predicted to bind with circRNA_0001006 and was reported to serve as a tumor suppressor in breast cancer. miR-424-5p was also involved in the function of many other circRNAs, such as the inhibition of gastric cancer cells by circ_LARP4, the promoter role of circ_RNF13 in HBV-hepatocellular carcinoma, and the enhancement of lung cell inflammation by cicr_0003420 [11–13]. Therefore, circRNA_0001006 might regulate the development of TNBC, and whether miR-424-5p mediates the function of circ_0001006 was also estimated.

## Results

### Expression and significance of circ_0001006 in TNBC

**Fig. 1:**
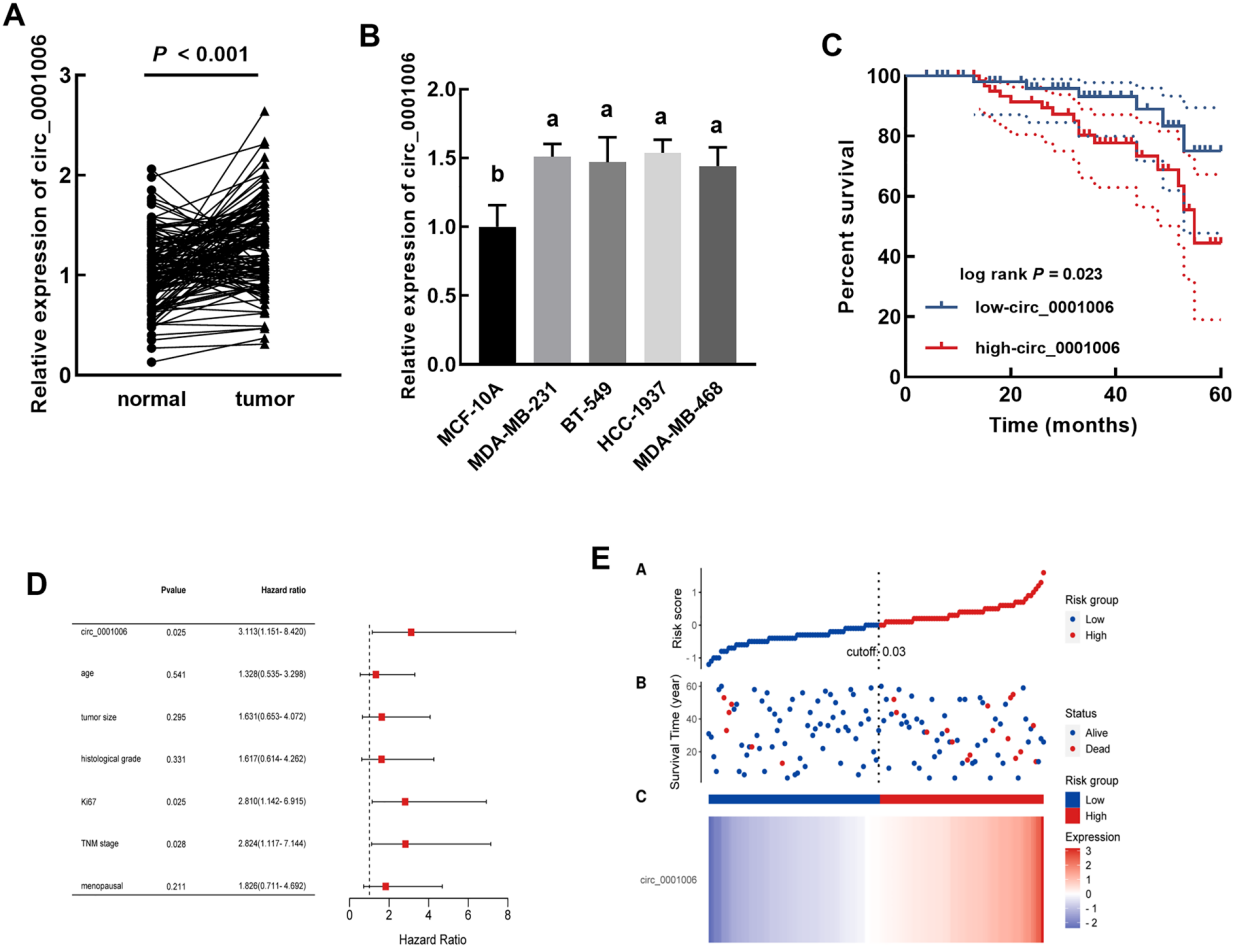
Expression and significance of circ_0001006 in TNBC. **A**, **B** Significant upregulation of circ_0001006 was observed in tumor tissues (**A**) and TNBC cells (**B**) compared with normal samples. **C**–**E** circ_0001006 was negatively correlated with TNBC patients’ overall survival (**C**) and served as an independent prognostic indicator (**D**). Increasing circ_0001006 expression indicated the increasing risk of TNBC patients (**E**). Different letters indicate significant differences (*P* < 0.05)

Significantly increasing circ_0001006 was observed in TNBC tumor tissues compared to the normal tissues (Fig. 1A). Consistently, circ_0001006 was also upregulated in TNBC cells relative to the normal cell (Fig. 1B).

Patients were grouped into the low-circ_0001006 and the high-circ_0001006 groups with their average expression in tissues as the cutoff. The higher expression of circ_0001006 was found to be associated with the higher histological grade (*P* = 0.005), Ki67 level (*P* < 0.001), and advanced TNM stage (*P* = 0.015) of patients (Table 1). miR-424-5p also showed significant association with TNBC patients’ histological grade (*P* = 0.028), Ki67 level (*P* = 0.008), and TNM stage (*P* = 0.011). Additionally, the high-circ_0001006 group showed a worse overall survival than that of the high-circ_0001006 group (Fig. 1C).

**Table 1:** The Association of circ_0001006 with patients’ clinicopathological features

Variant	Cases (n = 133)	Circ_0001006		*P*	miR-424-5p		*P*
		Low (n = 62)	High (n = 71)		Low (n = 70)	High (n = 63)	
Age				0.917			0.784
< 50	68	32	36		35	33	
≥ 50	65	30	35		35	30	
Tumor size				0.206			0.272
< 2	63	33	30		30	33	
≥ 2	70	29	41		40	30	
Histological grade				0.005			0.028
I–II	91	50	41		42	49	
III	42	12	30		28	14	
Ki67				< 0.001			0.008
< 20%	64	41	23		26	38	
≥ 20%	69	21	48		44	25	
TNM stage				0.015			0.011
I–II	82	45	37		36	46	
III	51	17	34		34	17	
Menopausal				0.790			0.927
Pre	66	30	36		35	31	
Post	67	32	35		35	32	

Circ_0001006 was identified as an independent prognostic indicator with the HR value of 3.113 (95% CI 1.151–8.420) together with Ki67 (HR = 2.810, 95% CI 1.142–6.915) and TNM stage (HR = 2.824, 95% CI 1.117–7.144, Fig. 1D). The risk scores of enrolled patients were increased with the increased expression of circ_0001006, which is consistent with the Kaplan-Meier and Cox analyses (Fig. 1E).

### Effect of circ_0001006 on TNBC cells

**Fig. 2:**
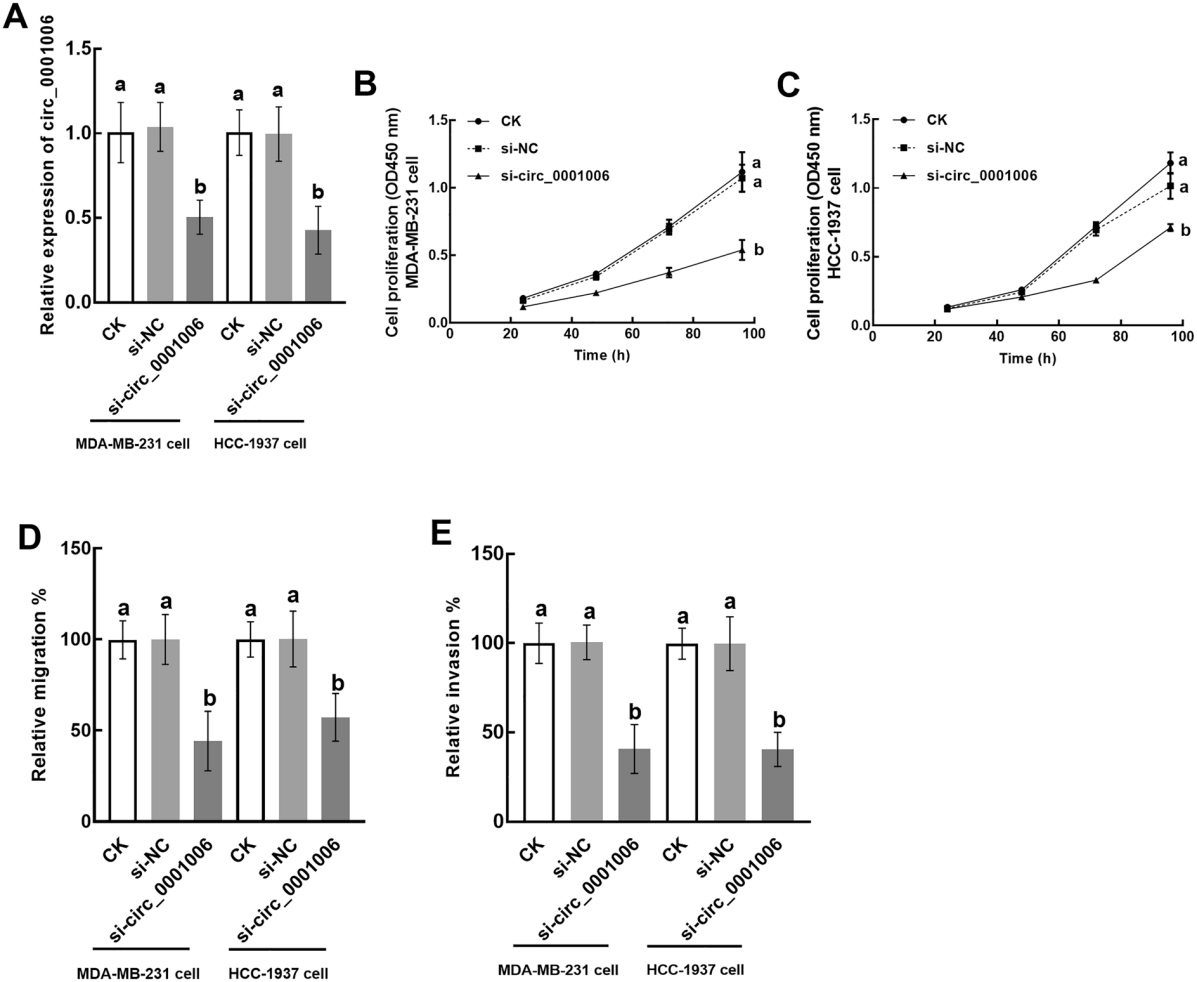
The function of circ_0001006 in TNBC cellular processes. **A** circ_0001006 was suppressed by the transfection of siRNA in MDA-MB-231 and HCC-1937 cells. **B**–**E** Silencing circ_0001006 dramatically inhibited the proliferation (**B**, **C**), migration (**D**), and invasion (**E**) of MDA-MB-231 (**B**) and HCC-1937 (**C**) cells. Different letters indicate significant differences (*P* < 0.05)

Circ_0001006 was suppressed by siRNA transfection in MDA-MB-231 and HCC-1937 cells compared with the cells without transfection or with negative controls (Fig. 2A). The knockdown of circ_0001006 inhibited the proliferation of both MDA-MB-231 (Fig. 2B) and HCC-1937 cells (Fig. 2C). Meanwhile, inhibition was also observed in the migration (Fig. 2D) and invasion (Fig. 2E) of MDA-MB-231 and HCC-1937 cells by circ_0001006 silencing.

### Circ_0001006 regulates miR-424-5p

**Fig. 3:**
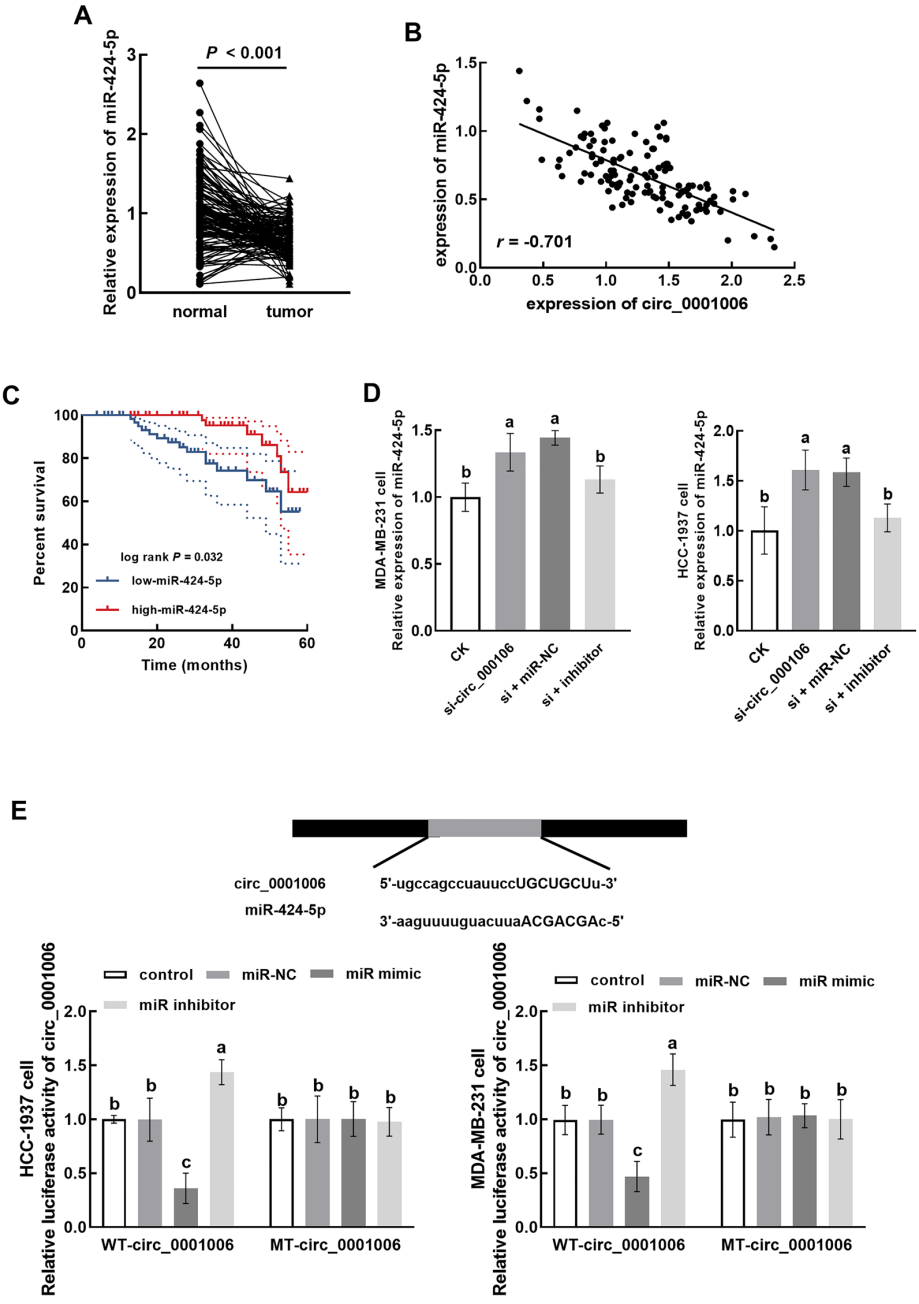
Interaction between circ_0001006 and miR-424-5p. **A**, **B** miR-424-5p was downregulated in tumor tissues (**A**) and showed a negative correlation with circ_0001006 (**B**). **C** Lower expression of miR-424-5p was significantly associated with the poorer overall survival of TNBC patients. **D** The knockdown of circ_0001006 suppressed the expression of miR-424-5p, which was reversed by the transfection of its inhibitor in MDA-MB-231 and HCC-1937 cells. **E** miR-424-5p negatively regulated the luciferase activity of circ_0001006 in MDA-MB-231 and HCC-1937 cells. Different letters indicate significant differences (*P* < 0.05)

miR-424-5p was significantly downregulated in TNBC tumor tissues compared with normal tissues (Fig. 3A), which showed a negative correlation with circ_0001006 in tumor tissues (r = − 0.701, Fig. 3B). The lower miR-424-5p expression was significantly correlated with poorer survival of TNBC patients (Fig. 3C). miR-424-5p was enhanced by silencing circ_0001006 in MDA-MB-231 and HCC-1937 cells, which was reversed by its inhibitors (Fig. 3D).

circ_0001006 was predicted to bind with miR-424-5p with several binding sites. miR-424-5p overexpression was found to significantly inhibit the luciferase activity of circ_0001006 and the knockdown of miR-424-5p showed an opposite effect (Fig. 3E).

### circ_0001006 regulates TNBC cell progression via miR-424-5p

**Fig. 4:**
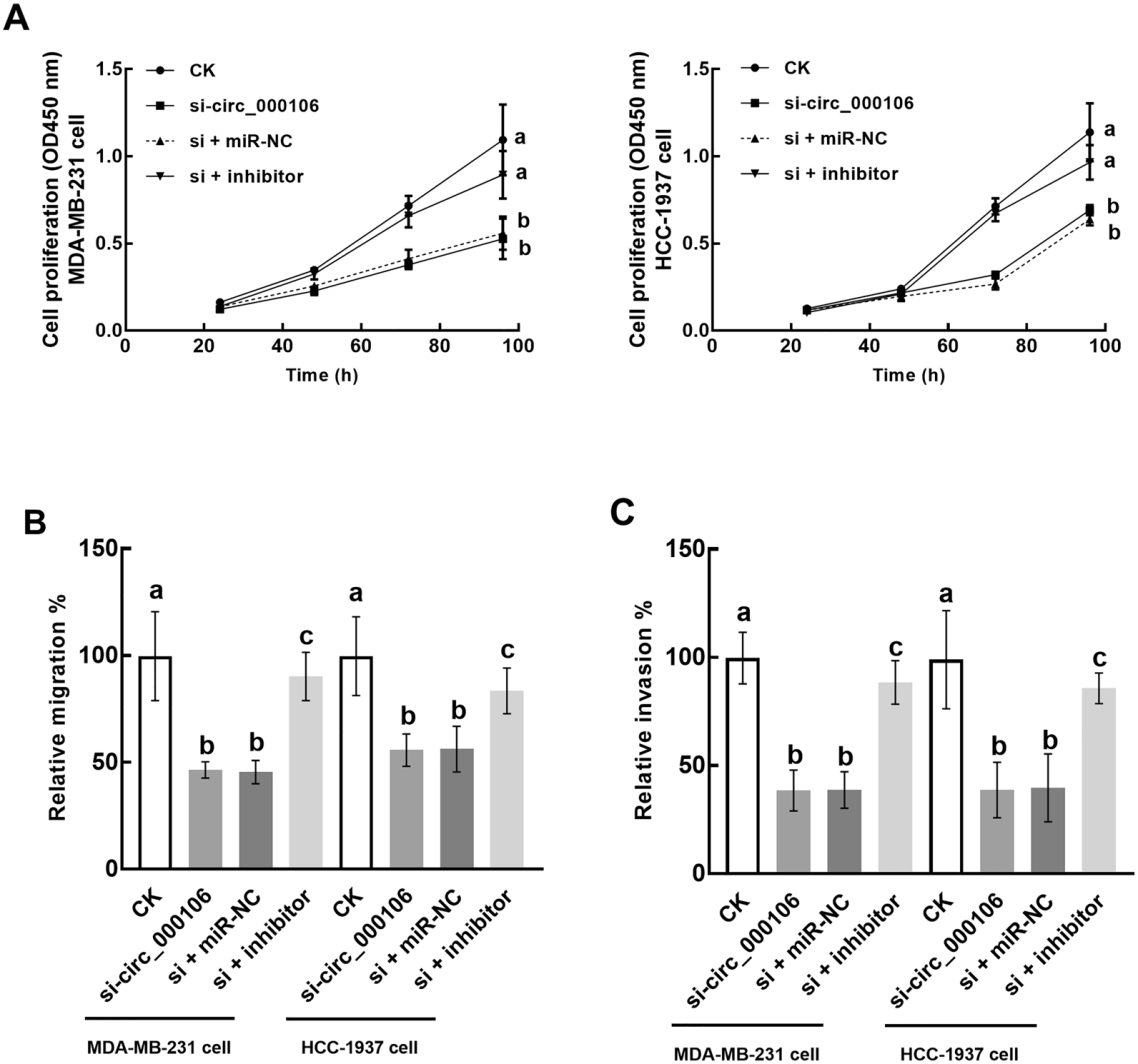
miR-424-5p mediates the effect of circ_0001006 on TNBC cellular processes. **A**–**C** The inhibitory effect of circ_0001006 knockdown on the proliferation (**A**), migration (**B**), and invasion (**C**) of MDA-MB-231 and HCC-1937 cells was alleviated by the suppression of miR-424-5p. Different letters indicate significant differences (*P* < 0.05)

The inhibitory effect of circ_0001006 on cell growth and metastasis of TNBC was dramatically alleviated by the suppression of miR-424-5p, indicating its involvement in mediating the function of circ_0001006 (Fig. 4A–C).

## Discussion

Due to the closed structure, circRNAs are not easily digested by exonucleases and therefore showed higher stability than other ncRNAs. Thanks to the development of sequencing technology and bioinformatics methods, the function of circRNAs in cellular processes and disease progression have attracted special attention. Several circRNAs were reported to regulate the tumorigenesis and progression of breast cancer. For example, circ_0005273 was identified as a tumor promoter that facilitated tumorgenesis and was considered a potential therapeutic target [14]. Downregulated circ_0053063 in breast cancer inhibited cell viability and proliferation via negatively modulating the miR-330-3p/PDCD4 axis [15]. Previously, Fu et al. established a circRNA profile of breast cancer brain metastasis by comparing the circRNA expression profile between breast cancer with or without brain metastasis, where circ_0001006 was found to be dysregulated. The abnormal expression of circ_0001006 was also observed in a plasma circRNA profiling in gastric cancer [16]. Moreover, circ_0001006 was also reported to aggravate cardiac hypertrophy with significant upregulation in transverse aortic constriction-induced mice [17]. In the present study, the expression of circ_0001006 was analyzed in TNBC patients, and its significant upregulation was observed in the tumor tissues. Increased circ_0001006 was correlated with patients’ higher Ki67 and histological grade and advanced TNM stage, which could indicate the malignant progression of TNBC. Additionally, the 5-year follow-up survey showed that the higher expression of circ_0001006, the poorer the survival time of TNBC patients is. Circ_0001006 was also identified as an independent prognostic indicator of TNBC, where the higher circ_0001006 indicated a higher risk. These clinical results revealed the involvement of circ_0001006 in TNBC development and suggested its potential in acting as a therapeutic target.

Increasing evidence reported the regulatory effect of circRNAs on the proliferation, apoptosis, metastasis, and other cellular processes in malignant tumors. Circ_0008039 possesses a consistent expression trend with circ_0001006 in breast cancer, and its knockdown significantly suppressed cell growth and metastasis [18]. In TNBC, circCD44 was also found to be upregulated, which promoted cell growth, metastasis, and tumorigenesis [19]. Herein, circ_0001006 was knockdown in TNBC cells, which inhibited cell proliferation, migration, and invasion, indicating circ_0001006 might serve as a tumor promoter in TNBC.

circRNAs could sponge miRNAs and indirectly regulate the expression and function of downstream genes to display their function. Circ_0001006 was demonstrated to sponge the miR-214-3p/PAK6 axis to perform its promoted effect on the development of cardiac hypertrophy [17]. The negative regulatory effect of circ_0001006 on miR-424-5p was revealed in TNBC cells in this study. miR-424-5p was reported to act as a tumor suppressor in ovarian cancer and regulate the sensitivity of ovarian cancer cells to erastin [20]. miR-424-5p was also demonstrated to participate in the development of endometrial cancer, colorectal cancer, and liver cancer [21–23]. Additionally, miR-424-5p was also suggested to mediate the function of circRNAs and lncRNAs in human cancers, such as the promoter role of circRNA ACTN4 in cholangiocarcinoma and the promoted effect of lncRNA MYLK-AS1 in hepatocellular carcinoma [24, 25]. In breast cancer, miR-424-5p was disclosed to suppress the viability and facilitate the chemosensitivity of breast cancer cells [26, 27] Here, miR-424-5p was found to reverse the inhibitory effect of circ_0001006 knockdown on cell growth and metastasis of TNBC cells, indicating the involvement of miR-424-5p in the regulation of TNBC cellular process by circ_0001006.

However, there are still limitations of this study needing to be improved in future investigations. Firstly, the clinical samples were collected from a single center with a relatively small sample size, which decrease the representativeness of the clinical results. The biological function of circ_0001006 was evaluated with a knockdown in TNBC cells. It is also necessary to assess the effect of circ_0001006 overexpression on TNBC cellular processes. But the present regular overexpression vector is not accurate enough for the transfection of siRNA, and the amount of transfection also needs to be quantified [28]. Therefore, further studies would focus on enlarging the sample size and improving the accuracy and efficacy of cell transfection.

## Conclusions

In conclusion, upregulated circ_0001006 in TNBC indicated malignant development and poor prognosis of TNBC patients. Silencing circ_0001006 suppressed cell growth and metastasis of TNBC via negatively regulating miR-424-5p.

## Materials and methods

### Study subjects

This study was performed in line with the principles of the Declaration of Helsinki. Approval was granted by the Ethics Committee of Zibo Central Hospital. There were 133 TNBC patients enrolled in the present study from 2014 to 2016 at Zibo Central Hospital. The inclusion and exclusion criteria are as follows:

1) Women patients are primarily diagnosed with TNBC according to cell or histopathology and imaging examinations.

2) Patients without a history of radiotherapy or chemotherapy.

3) Patients without any serious complications, including but not limited to serious cardiovascular and cerebrovascular diseases, hematopoietic system disorders, liver and kidney dysfunction, and other malignant tumors.

4) Patients with completed medical records and laboratory results.

5) Patients or their immediate families were aware of the study process and signed the informed consent.

6) Women who are pregnant or breastfeeding were excluded.

7) Patients lost to follow-up were excluded.

### Sample collection

Tissue samples were collected during surgery including tumor and normal para-cancerous tissues meeting the detection requirements. Collected tissues were confirmed by at least two pathologists and reached an agreement. Tissues were frozen in liquid nitrogen and stored at − 80 °C after marking until analyses.

### Follow-up survey

All patients were followed up after surgery for 3–60 months to track their recovery and survival status. Recurrence, metastasis, and TNBC-related deaths were considered as the ending events of the follow-up survey.

### Cell culture

Human-sourced TNBC cell lines (MDA-MB-231, BT-549, HCC-1937, and MDA-MB-468) and a normal human breast cell (MCF-10 A) were purchased from ATCC. Cells were maintained in the DMEM culture medium at 37 °C with 5% CO_2_. The logarithmic phase cells were used for the subsequent experiments.

### Cell transfection

Lipofectamine 2000 (Invitrogen, USA) and siRNA were mixed and bleeding with an FBS-free culture medium. Cells were seeded in the 24-well plates supplied with the transfection mixture and incubated for 6 h, and then the substrate was replaced with a completed culture medium. Cells were available after 48 h of cell transfection.

### Total RNA extraction

Cells mixed with TRIzol reagent (Invitrogen, USA) for 5 min after washing with PBS three times to isolate total RNA. For tissue samples, tissues were mixed with TRIzol reagent on ice and cut up. The homogenate was prepared by ultrasound for 5 min. Then, the mixture was treated with chloroform and centrifugated at 12,000*g* for 15 min. The supernatant was mixed with isopropyl alcohol and total RNA was obtained after centrifugation at 12,000*g* for 15 min. The purification and concentration were analyzed by the value of OD260/280. OD260/280 ranged 1.8-2.0 and concentration over 40 µg/mL indicated the high-quality RNA.

### Real-time quantitative PCR

The extracted total RNA was reversed to cDNA on the ice with the Transcriptor First Strand cDNA Synthesis Kit (for circ_0001006, Roche, Indian) and TaqMan MiR Reverse Transcription Kit (for miR-424-5p, Thermo, USA). Then, cDNA was used for the quantitation of circ_0001006 and miR-424-5p using SYBR qPCR Mix (Toyobo, Japan) on the qRT-PCR system (Thermo, USA). The reaction was performed with 5 cycles as follows: cycle 1, 95 °C for 30 s; cycle 2, 95 °C for 5 s, 60 °C for 34 s of 40 cycles; cycle 3, 95 °C for 10 s; cycle 4, 95 °C for 1 min; cycle 5, 97 °C for 1 s. The relative expression levels were calculated with the 2^−△△CT^ method normalized to GAPDH (for circ_0001006) and cel-miR-39 (for miR-424-5p).

### Cell proliferation assay

Cells were seeded in the 96-well plates and incubated with the completed culture medium for 24, 48, 72, and 96 h. Then, the culture medium was discarded and the CCK8 reagent was added. A 4-h incubation was carried out at 37 °C, and then OD450 was measured.

### Cell metastasis assay

Cells were seeded in the upper chamber of Transwell plates supplied with FBS-free culture medium, and the lower chamber was filled with the completed chamber. The plates were incubated for 24 h. Then, the culture medium in the lower chamber was replaced with 4% paraformaldehyde and foxed for 30 min. After washing with PBS, cells were stained with 0.1% crystal violet for 15 min at room temperature and counted with an optical light inverted microscope (Olympus, USA).

### Dual-luciferase reporter assay

The wild-type and mutant-type circ_0001006 reporter vectors were constructed with the binding sites and mutant sites, respectively. Cells were seeded in the 12-well plates and co-transfected with reporter vectors and miR-424-5p mimic or inhibitor. The relative luciferase activity was measured using a microplate Reader and normalized to Renilla.

### Statistical analyses

Experimental data were presented as mean ± SD (n = 3) and analyzed by one-way ANOVA with Turkey’s post-hoc test using Graphpad Prism 9.3 (*P* < 0.05). The clinical characteristics of enrolled patients were analyzed by Chi-square test. The prognosis information was analyzed with Kaplan-Meier and Cox regression analysis using SPSS26.0 software. The comparison between normal and tumor samples was carried out with Student’s t-test, while the Spearman correlation analysis was performed to assess the correlation between circ_0001006 and miR-424-5p expression.

## Data Availability

The datasets used and/or analysed during the current study are available from the corresponding author on reasonable request.

## References

[1] DeSantis CE, Ma J, Goding Sauer A, Newman LA, Jemal A (2017). Breast cancer statistics, 2017, racial disparity in mortality by state. CA Cancer J Clin.

[2] Anastasiadi Z, Lianos GD, Ignatiadou E, Harissis HV, Mitsis M (2017). Breast cancer in young women: an overview. Updates Surg.

[3] Howard FM, Olopade OI (2021). Epidemiology of Triple-Negative breast Cancer: a review. Cancer J (Sudbury Mass).

[4] Kumar P, Aggarwal R (2016). An overview of triple-negative breast cancer. Arch Gynecol Obstet.

[5] A MB RVS (2014). Breast cancer biomarkers: risk assessment, diagnosis, prognosis, prediction of treatment efficacy and toxicity, and recurrence. Curr Pharm Des.

[6] Chen L, Wang C, Sun H, Wang J, Liang Y, Wang Y (2021). The bioinformatics toolbox for circRNA discovery and analysis. Brief Bioinform.

[7] Zhou WY, Cai ZR, Liu J, Wang DS, Ju HQ, Xu RH (2020). Circular RNA: metabolism, functions and interactions with proteins. Mol Cancer.

[8] Xu X, Zhang J, Tian Y, Gao Y, Dong X, Chen W (2020). CircRNA inhibits DNA damage repair by interacting with host gene. Mol Cancer.

[9] Zang J, Lu D, Xu A (2020). The interaction of circRNAs and RNA binding proteins: an important part of circRNA maintenance and function. J Neurosci Res.

[10] Fu B, Zhang A, Li M, Pan L, Tang W, An M (2018). Circular RNA profile of breast cancer brain metastasis: identification of potential biomarkers and therapeutic targets. Epigenomics.

[11] Zhang J, Liu H, Hou L, Wang G, Zhang R, Huang Y (2017). Circular RNA_LARP4 inhibits cell proliferation and invasion of gastric cancer by sponging mir-424-5p and regulating LATS1 expression. Mol Cancer.

[12] Chen Y, Li S, Wei Y, Xu Z, Wu X (2021). Circ-RNF13, as an oncogene, regulates malignant progression of HBV-associated hepatocellular carcinoma cells and HBV infection through ceRNA pathway of circ-RNF13/miR-424-5p/TGIF2. Bosn J Basic Med Sci.

[13] Yang H, Zhang C, Zhao Z (2022). Lipopolysaccharide-induced lung cell inflammation and apoptosis are enhanced by circ_0003420/miR-424-5p/TLR4 axis via inactivating the NF-kappaB signaling pathway. Transpl Immunol.

[14] Wang X, Ji C, Hu J, Deng X, Zheng W, Yu Y (2021). Hsa_circ_0005273 facilitates breast cancer tumorigenesis by regulating YAP1-hippo signaling pathway. J Exp Clin Cancer Res.

[15] Ji C, Hu J, Wang X, Zheng W, Deng X, Song H (2021). Hsa_circ_0053063 inhibits breast cancer cell proliferation via hsa_circ_0053063/hsa-miR-330-3p/PDCD4 axis. Aging.

[16] Li T, Shao Y, Fu L, Xie Y, Zhu L, Sun W (2018). Plasma circular RNA profiling of patients with gastric cancer and their droplet digital RT-PCR detection. J Mol Med (Berl).

[17] Lin X, Zhang L, Zhang W, Lei X, Lu Q, Ma A (2022). Circular RNA circ_0001006 aggravates cardiac hypertrophy via miR-214-3p/PAK6 axis. Aging.

[18] Dou D, Ren X, Han M, Xu X, Ge X, Gu Y (2021). Circ_0008039 supports breast cancer cell proliferation, migration, invasion, and glycolysis by regulating the miR-140-3p/SKA2 axis. Mol Oncol.

[19] Li J, Gao X, Zhang Z, Lai Y, Lin X, Lin B (2021). CircCD44 plays oncogenic roles in triple-negative breast cancer by modulating the miR-502-5p/KRAS and IGF2BP2/Myc axes. Mol Cancer.

[20] Ma LL, Liang L, Zhou D, Wang SW (2021). Tumor suppressor mir-424-5p abrogates ferroptosis in ovarian cancer through targeting ACSL4. Neoplasma.

[21] Dai W, Zhou J, Wang H, Zhang M, Yang X, Song W (2020). Mir-424-5p promotes the proliferation and metastasis of colorectal cancer by directly targeting SCN4B. Pathol Res Pract.

[22] Ning D, Chen J, Du P, Liu Q, Cheng Q, Li X (2021). The crosstalk network of XIST/miR-424-5p/OGT mediates RAF1 glycosylation and participates in the progression of liver cancer. Liver Int.

[23] Wang P, Liu T, Zhao Z, Wang Z, Liu S, Yang X (2021). SPTBN2 regulated by mir-424-5p promotes endometrial cancer progression via CLDN4/PI3K/AKT axis. Cell death discovery.

[24] Chen Q, Wang H, Li Z, Li F, Liang L, Zou Y (2022). Circular RNA ACTN4 promotes intrahepatic cholangiocarcinoma progression by recruiting YBX1 to initiate FZD7 transcription. J Hepatol.

[25] Teng F, Zhang JX, Chang QM, Wu XB, Tang WG, Wang JF (2020). LncRNA MYLK-AS1 facilitates tumor progression and angiogenesis by targeting miR-424-5p/E2F7 axis and activating VEGFR-2 signaling pathway in hepatocellular carcinoma. J Exp Clin Cancer Res.

[26] Dastmalchi N, Safaralizadeh R, Hosseinpourfeizi MA, Baradaran B, Khojasteh SMB (2021). MicroRNA-424-5p enhances chemosensitivity of breast cancer cells to Taxol and regulates cell cycle, apoptosis, and proliferation. Mol Biol Rep.

[27] Dastmalchi N, Hosseinpourfeizi MA, Khojasteh SMB, Baradaran B, Safaralizadeh R (2020). Tumor suppressive activity of mir-424-5p in breast cancer cells through targeting PD-L1 and modulating PTEN/PI3K/AKT/mTOR signaling pathway. Life Sci.

[28] Zhao W, Dong M, Pan J, Wang Y, Zhou J, Ma J (2019). Circular RNAs: a novel target among noncoding RNAs with potential roles in malignant tumors (review). Mol Med Rep.

